# The clinical value of the combined detection of circulating low ⁃ density neutrophils and inflammatory markers in breast cancer with lymph node metastasis

**DOI:** 10.1186/s12885-026-16384-6

**Published:** 2026-06-23

**Authors:** Dongping Mo, Cong Li, Xuelian Mao, Lijun Zheng, Feng Yan

**Affiliations:** 1 Department of Clinical Laboratory, Jiangsu Cancer Hospital & Nanjing Medical University Affiliated Cancer Hospital & Jiangsu Institute of Cancer Research, Nanjing, Jiangsu 210009 People’s Republic of China; 2 Department of Clinical Laboratory, Nanjing Lishui District Hospital of Traditional Chinese Medicine, Nanjing, People’s Republic of China

**Keywords:** Low ⁃ density neutrophils, Inflammatory markers, Lymph node metastasis, Breast cancer, Nomogram model

## Abstract

**Background:**

Breast cancer (BC) is the most prevalent cancer in women globally, with axillary lymph node metastasis (LNM) and distant metastasis being key prognostic factors linked to survival. Circulating low-density neutrophils (LDNs) in cancer patients are associated with tumor-supportive role. This study assessed the clinical relevance of combining circulating LDNs with hematological indicators in BC patients with LNM.

**Methods:**

Peripheral blood samples from 184 BC patients were analyzed using flow cytometry to measure the LDNs levels. Lasso logistic regression was used for variable selection, and univariate and multivariate logistic regression identified independent risk factors. A predictive nomogram was developed and validated with an external cohort.

**Results:**

LDNs were elevated in BC patients, especially those with LNM. Seven variables—LDNs, CEA, CA153, PLR, SIRI, NMLR, and NHR—were identified for further analysis. Multivariate logistic regression identified five of these as independent risk factors for LNM: LDNs (OR = 2.307, *P* = 0.032), CEA (OR = 4.027, *P* < 0.001), CA153 (OR = 2.440, *P* = 0.016), PLR (OR = 2.563, *P* = 0.014), and SIRI (OR = 4.617, *P* = 0.003). The nomogram model based on these factors demonstrated good predictive performance with an optimism-corrected C-statistic of 0.780, consistent in the external validation set (AUC = 0.762). The calibration curve confirmed good clinical applicability.

**Conclusion:**

This study suggests circulating LDNs levels may be potential biomarkers for BC progression, and a nomogram combing LDNs with other hematological indicators shows potential for identifying patients with LNM.

**Supplementary Information:**

The online version contains supplementary material available at https://doi.org/10.1186/s12885-026-16384-6.

## Introduction

Breast cancer is the most common cancer among women, representing about 23.8% of new cases and 15.4% of female cancer deaths [1]. Lymph node status is crucial for determining the stage, grade, treatment, and prognosis of breast cancer, with sentinel lymph node (SLN) status guiding axillary lymph node treatment [2]. Patients with axillary lymph node metastasis (LNM) exhibit a significantly lower 5-year overall survival rate than those without LNM. Although pathological biopsy remains the clinical gold standard for LNM diagnosis, SLN biopsy is associated with complications such as limited shoulder abduction, paresthesia, arm numbness, infection, and seroma [3]. Furthermore, as an invasive procedure, it may result in overtreatment for patients ultimately found to be SLN-negative. Therefore, it is crucial to identify practical, less invasive, and readily available indicators to enable earlier and effective diagnosis of LNM.

Neutrophils, comprising 50%–70% of peripheral leukocytes, are the most abundant immune cells in the human body. After maturing in the bone marrow, they enter the bloodstream to play a crucial role in innate immunity by defending against pathogens [4, 5]. Traditionally linked to innate immunity, neutrophils are now also associated with diseases like cancer due to their interaction with other immune cells [6]. Their heterogeneity allows for subclassification based on differentiation status, surface markers, or density [7]. Neutrophil subpopulation that has been studied the most and has achieved the most breakthrough research results is low-density neutrophils (LDNs). LDNs, originally described as low-density granulocytes (LDGs) by Hacbarth and Kajdacsy-Balla in 1986 [8], represent a distinct neutrophil subset that resides in the peripheral blood mononuclear cell (PBMC) fraction due to their unique buoyant density [9, 10]. Phenotypically, LDGs are identified as CD14^−^CD15⁺, a profile that enables their discrimination from monocytes within the PBMC fraction [11, 12].

LDNs exhibit typical neutrophil functions such as phagocytosis, chemotaxis and cytokine secretion but release higher levels of pro-inflammatory cytokines like IL-6 and IL-8, contributing to strong pro-inflammatory or immunosuppressive effects [13, 14]. An increasing number of studies suggest LDNs significantly contribute to the pathogenesis and progression of diseases, including autoimmune disorders, infections disorders, and cancer [15]. Patients with cancer have higher numbers of LDNs, which are thought to be primed neutrophils with an increased neutrophil extracellular traps (NETs) formation potential [16–18]. In advanced-stage lung cancer, patients with LDNs > 10% had significantly poorer survival (HR = 2.01; 95% CI: 0.975–4.533) [19]. Additionally, in head and neck cancer patients undergoing immunotherapy, higher levels of LDNs were correlated with significantly shorter progression-free survival (1.8 months vs. 10.9 months), indicating that LDNs may serve as a predictive biomarker for immune checkpoint inhibitors response [18]. Therefore, LDNs may represent promising biomarkers for prognostic monitoring in malignant tumors. Nevertheless, their utility in the clinical assessment of these malignancies remains to be elucidated.

Emerging evidence suggests a significant association between the pathophysiological mechanisms of breast cancer and the tumor inflammatory microenvironment [20, 21]. Inflammatory mediators are known to promote angiogenesis, enhance tumor cell survival, and facilitate the degradation of extracellular matrix components, thereby supporting tumor invasion and metastasis [22, 23]. Inflammatory markers derived from complete blood count (CBC) parameters, such as the neutrophil-to-lymphocyte ratio (NLR), platelet-to-lymphocyte ratio (PLR), systemic inflammation response index (SIRI), and systemic immune inflammation index (SII), have been shown to correlate with the progression of various tumors, including biliary tract cancer [24], non-small cell lung cancer [25, 26], and breast cancer [27]. Importantly, these biomarkers are advantageous due to their accessibility through routine blood tests in cancer patients. Nevertheless, little research has been conducted on the combined assessment of LDNs and CBC-derived inflammatory markers in breast cancer.

This study aimed to investigate whether LDNs levels are elevated in breast cancer patients and to explore their correlation with clinical characteristics. To our knowledge, this is the first study to simultaneously assess LDNs levels and hematological indicators from peripheral blood tests in breast cancer patients. Using Lasso and multivariate logistic regression, we identified relevant indicators and developed a nomogram. These findings may offer a new perspective for clinical LNM assessment in breast cancer and may have potential implications for patient prognosis.

## Materials and methods

### Subjects and samples

The study was conducted in accordance with the Declaration of Helsinki (revised in 2013) and approved by the Clinical Research Ethics Committee of Nanjing Medical University (No.2024 − 473). Individuals with a definite diagnosis of breast cancer at the Department of Breast Surgery of Jiangsu Cancer Hospital between October 2024 and June 2025 were included. Patients were included if they met the following criteria: (1) female, (2) age between 18 and 70 years; (3) primary breast cancer, (4) not receiving any anti-tumor treatment, including surgery, chemotherapy, and neoadjuvant chemotherapy, (5) no distant metastasis, (6) no background inflammatory diseases, and (7) no history of serious immune disorders or malignant hematologic disease. A total of 184 patients were included in this study, and 82 patients had LNM according to the American Joint Committee on Cancer (AJCC)/Union for International Cancer Control (UICC) 8th edition staging system. The basic characteristics of all the subjects are detailed in supplementary materials Table S1. Seventy healthy individuals without these diseases were recruited as controls. In addition, a temporally distinct external validation cohort was collected from the same institution, comprising 54 newly diagnosed breast cancer patients between March 16 and April 25, 2026. No patients overlapped between the two cohorts, and all participants met the aforementioned inclusion and exclusion criteria.

All samples were collected during routine clinical practice, and this collection did not influence the patient treatment or diagnosis.

### Isolation of peripheral blood mononuclear cells

Samples from patients before anticancer therapy. After the physical examination, the remaining EDTA-anticoagulated whole blood was collected. PBMCs were isolated using a Ficoll separation solution gradient as described previously [28, 29]. Briefly, each blood sample was diluted 1:1 in RPMI-1640. The mixture was then slowly added to the top of the Ficoll separation solution (Absin, abs 930 − 200 ml). After centrifugation at 2000 rpm for 20 min at room temperature without breaking, PBMCs (including LDNs) were carefully collected from the interface between the plasma layer and the Ficoll separation solution. Erythrocytes were lysed with red blood cell lysis buffer (Absin, abs 9101 − 100 ml) for 15 min at room temperature, washed with PBS, and centrifuged at 400 × g for 5 min for further analysis.

### Flow cytometry

Surface flow cytometry analyses were performed immediately after PBMCs fractions were obtained. Cells were resuspended in PBS and stained with the following fluorochrome-conjugated antibodies: CD45-PE-Cy7 (CEGER, Z020500201), CD14-PE (BD Pharmingen™, 555398), and CD15-APC (BD Pharmingen™, 551376). According to the protocols, PBMCs fractions were incubated with these antibodies in an amount of 20 µL for 20 min at room temperature in the dark, then washed and resuspended in PBS. Staining was evaluated by Agilent NovoCyte-D2060R. The gating strategy was as follows: Cells were first gated on FSC-A vs. SSC-A to exclude debris and dead cells (P1). Doublets were excluded by gating on FSC-A vs. FSC-H. Leukocytes were then gated as CD45⁺ cells. Within the CD45⁺ population, LDNs were identified as CD14⁻CD15⁺ cells. Although neutrophils are typically removed during PBMC isolation due to their high density, LDNs co-purify with the PBMC fraction because of their distinct buoyant properties [30]. The LDNs percentage was calculated as: (CD45⁺CD14⁻CD15⁺) / (total CD45⁺ cells) × 100%. The relative percentage of LDNs based on specific markers was quantified using NovoExpress software 1.5.6 (Agilent Technologies, Inc.).

### Clinical data calculation

Basic data and laboratory indicators within 24 h of admission were collected from the electronic medical record system of Jiangsu Cancer Hospital. The basic data included age, height, weight, menstrual history, smoking history, drinking history, and family history. Laboratory indicators included neutrophil count (NE), lymphocyte count (LY), monocyte count (MO), platelet count (Plt), albumin (Alb), high-density lipoprotein cholesterol (HDL­C), and the tumor markers CEA, CA153, and CA125. The calculation of peripheral blood-derived inflammatory markers was performed consistent with the methodology we previously employed, including NLR, PLR, lymphocyte-to-monocyte ratio (LMR), SII, SIRI, prognostic nutritional index (PNI), aggregate index of systemic inflammation (AISI), (neutrophil + monocyte)-to-lymphocyte ratio (NMLR), NHR, monocyte-to-HDL-C ratio (MHR), lymphocyte to HDL-C ratio (LHR), and platelet to HDL-C ratio (PHR) [31].

### Statistical analysis

All statistical analyses were performed using IBM SPSS Statistics Version 20.0, GraphPad Prism v9.4.1, and R version 4.2.1. Normality of data distribution was first assessed with the Kolmogorov–Smirnov test. Normally distributed continuous variables were described as mean ± standard deviation; otherwise, data were reported as medians (interquartile range [IQR]). Differences between groups were evaluated using the Student’s *t*-test or one-way ANOVA for normally distributed data, and the Mann–Whitney *U* or Kruskal–Wallis test for non-normally distributed data. Categorical variables were compared with the chi-square test. Lasso logistic regression model was fitted using the “glmnet” package (version 4.1.7). Parameter selection in the Lasso model used tenfold cross-validation via minimum criterion. Partial likelihood deviation (binomial deviation) curves and logarithmic (lambda) curves were plotted. Use the minimum standard and 1se (1-SE standard) of the minimum standard to draw a vertical dashed line at the optimal value. Correlations were examined using either Spearman rank correlation or Pearson correlation as appropriate. The efficacy of LDNs and other variables was evaluated based on the sensitivity, specificity, and ROC-AUC. Pearson’s chi-squared test was used to evaluate the association between the percentage of LDNs and the clinicopathological parameters. To analyze relative risks for patients with LNM, univariate and multivariate logistic regression analyses were performed, with odds ratios (OR) and 95% confidence intervals (CI) reported. Multicollinearity among the independent predictor was assessed using the variance inflation factor (VIF). A VIF value exceeding 5 was considered indicative of significant collinearity. A nomogram was constructed based on the multivariate logistic regression results. Finally, we verified the model using the independent external cohort. All statistical tests were two-sided, and a *P*-value < 0.05 was considered statistically significant.

## Results

### Low-density neutrophils are expanded in patients with breast cancer

**Fig. 1:**
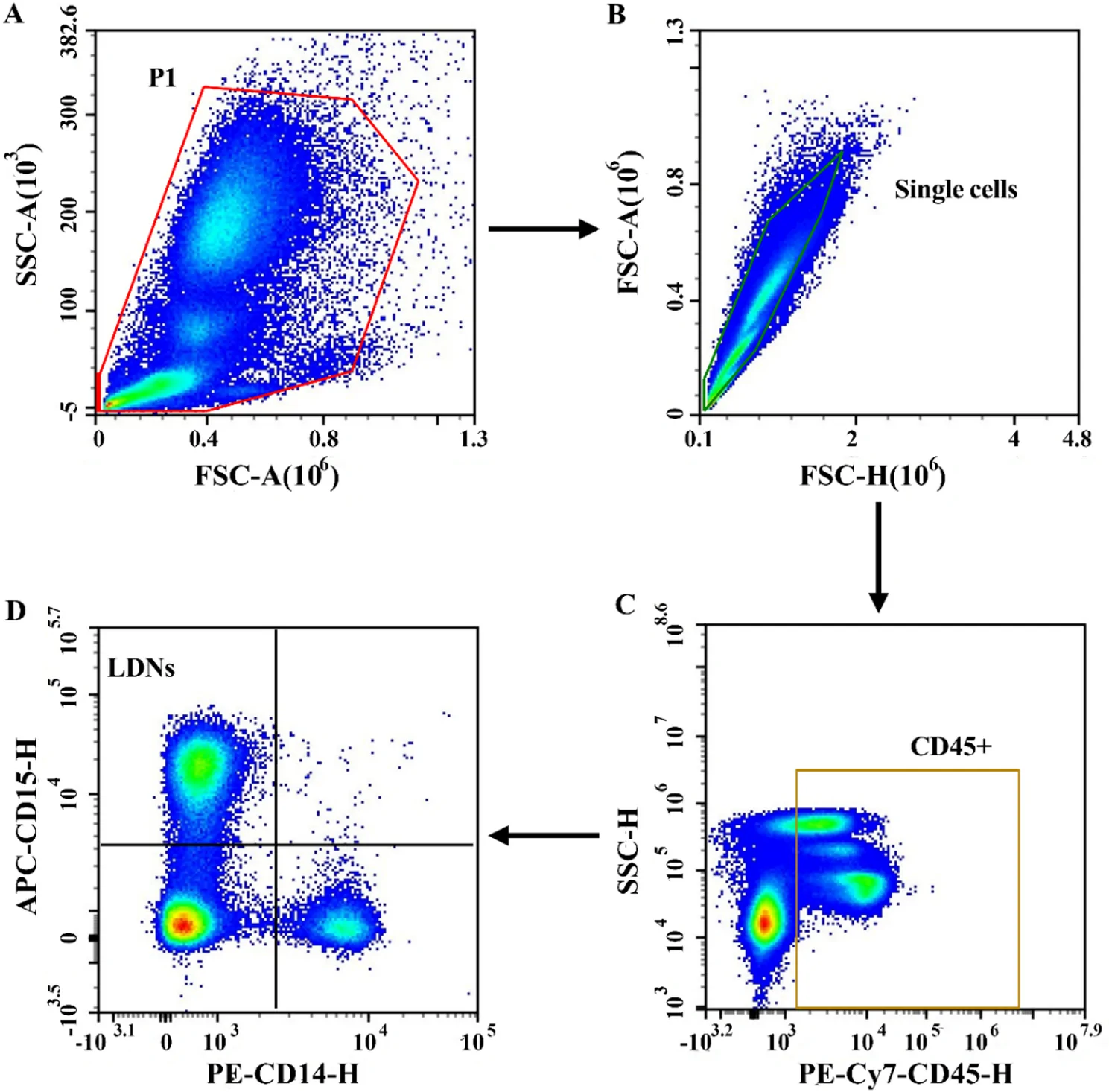
Representative flow cytometry gating strategy for the identification of low-density neutrophils. The gating strategy was as follows: (**A**) Cell population, PBMCs were gated on FSC-A vs. SSC-A to exclude debris and dead cells (P1); (**B**) Single cells, Doublets were excluded by gating on FSC-A vs. FSC-H; (**C**) Leukocytes, Leukocytes were gated as CD45⁺ cells; (**D**) LDN identification, Within the CD45⁺ population, LDNs were identified as CD14⁻CD15⁺ cells

**Fig. 2:**
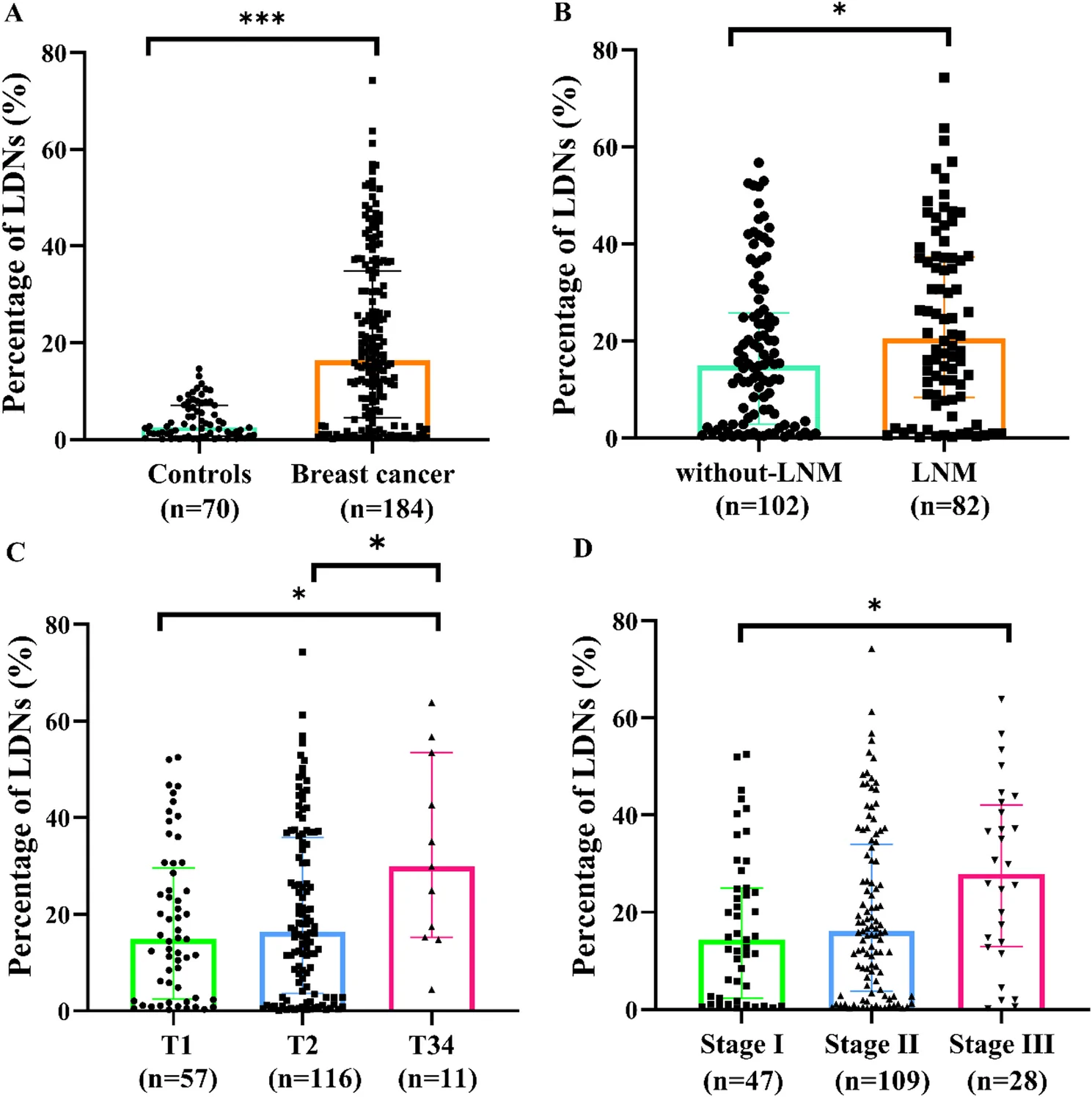
LDNs percentage in different patient groups. (**A**) Comparison between healthy controls and breast cancer patients; (**B**) Comparison between patients without LNM or with LNM; (**C**) Distribution across T stages (T1, T2, T3–T4); (**D**) Distribution across TNM stages (Stage I, II, III). *P < 0.05, ***P < 0.001

PBMCs were isolated from the peripheral blood of all subjects using density gradient centrifugation. The percentage of LDNs was detected via flow cytometry (Fig. 1**).** The analysis revealed that the percentage of LDNs was 6.6-fold higher in breast cancer patients (16.43% [4.75%, 34.63%]) compared to healthy controls (2.49% [0.80%, 6.99%], *P* < 0.001). Furthermore, the percentage of LDNs was markedly elevated in patients with LNM compared to those without LNM [20.53% (8.64%, 37.16%) vs. 15.00% (2.80%, 25.47%), *P* = 0.035] (Fig. 2A, B). Notably, when breast cancer patients were stratified according to T stage, a progressive increase in the percentage of LDNs was observed, although this trend did not reach statistical significance (median 14.89%, 16.43%, and 29.87%, respectively; *P* = 0.064). However, the percentage of LDNs in patients with T3-4 stage was significantly higher than in those with T1 (*P* = 0.016) and T2 (*P* = 0.039) stage (Fig. 2C). Interestingly, when patients were categorized based on TNM staging, a significant progressive increase in the percentage of LDNs was observed (median 14.43%, 16.11%, and 27.90%, respectively; *P* = 0.046) (Fig. 2D).

### Baseline characteristics of patients according to status of LNM

Table 1 shows that 82 breast cancer patients (44.57%) had pathologically confirmed LNM. Compared to the non-LNM group, significantly higher median levels were observed for LDNs, CEA, CA153, NLR, PLR, SII, SIRI, AISI, NMLR, and NHR (all *P* < 0.05). In contrast, age, BMI, other hematological indicators (CA125, LMR, PNI, MHR, LHR, and PHR), and the positive rates of ER, PR, HER-2 and Ki-67 were not significantly different between the two groups (all *P* > 0.05).

**Table 1:** Comparison of clinicopathological characteristics in breast cancer patients with or without lymph node metastasis

Characteristics	No lymph node metastasis (*n* = 102)	Lymph node metastasis (*n* = 82)		*P* value
Age(years)	53(42, 59)	51(46, 56)		0.527
BMI (kg/m^2^)	24.39(21.84, 26.33)	24.18(22.27, 27.02)		0.629
LDNs (%)	15.00(2.80, 25.47)	20.53(8.64, 37.16)		0.035*
CEA (ng/mL)	1.30(0.86, 1.96)	1.71(1.18, 2.42)		0.004**
CA153 (U/mL)	9.14(6.58, 12.80)	11.90(8.33, 18.80)		0.002**
CA125 (U/mL)	9.87(6.85, 12.45)	9.89(7.74, 12.78)		0.400
NLR	1.61(1.21, 1.89)	1.83 (1.48, 2.46)		0.002**
PLR	117.68(98.71, 138.61)	130.50(107.71, 162.92)		0.049*
LMR	4.78(3.92, 5.75)	4.54(3.74, 5.35)		0.173
SII	354.55(268.69, 446.76)	423.33(317.69, 524.74)		0.002**
PNI	54.10(50.86, 57.36)	53.15(50.11, 57.03)		0.415
SIRI	0.55(0.44, 0.85)	0.71(0.59, 0.85)		0.007**
AISI	141.10(86.55, 181.47)	163.57(126.59, 220.02)		0.015*
NMLR	1.80(1.40, 2.11)	2.04(1.72, 2.67)		0.002**
NHR	2.16(1.54, 2.96)	2.43(1.85, 3.31)		0.019*
MHR	0.29(0.22, 0.39)	0.32(0.24, 0.39)		0.087
LHR	1.42(1.02, 1.75)	1.32(1.05, 1.86)		0.877
PHR	167.36(132.79, 205.23)	177.79(140.45, 229.20)		0.093
ER				0.542
Negative	30(29.41)	20(24.39)		
Positive	70(68.63)	61(74.39)		
Unknown	2(1.96)	1(1.22)		
PR				0.385
Negative	38(37.25)	34(41.46)		
Positive	60(58.82)	47(57.32)		
Unknown	4(3.93)	1(1.22)		
Her-2				0.831
Negative	13(12.75)	10(12.19)		
Positive	86(84.31)	71(86.59)		
Unknown	3(2.94)	1(1.22)		
Ki-67				0.910
≤ 30%	45(44.12)	36(43.90)		
> 30%	54(52.94)	43(52.44)		
Unknown	3(2.94)	3(3.66)		

*BMI* body mass Index, *LDNs* low ⁃ density neutrophils, *CEA* carcinoembryonic antigen, *CA125* carbohydrate antigen 125, *CA153* carbohydrate antigen 153, *NLR* neutrophil-to-lymphocyte ratio; *PLR:* platelet-to- lymphocyte ratio, *LMR* lymphocyte-to-monocyte ratio, *SII* systemic immune- inflammation index, *PNI* prognostic nutritional index, *SIRI* the systemic inflammation response index, *AISI* aggregate index of systemic inflammation, *NMLR* (neutrophil + monocyte)-to-lymphocyte ratio, *NHR* neutrophil to HDL­C ratio, *MHR* monocyte to HDL­C ratio, *LHR* lymphocyte to HDL-C ratio, *PHR* platelet to HDL-C ratio, *ER* estrogen receptor, *PR* progesterone Receptor, *Her-2* epidermal growth factor receptor-2. The measurement data were expressed as the median and quartile (25%, 75%), and the enumeration data were expressed as frequency and rate (%), *P < 0.05, **P < 0.01

### Variable screening through Lasso logistic regression

**Fig. 3:**
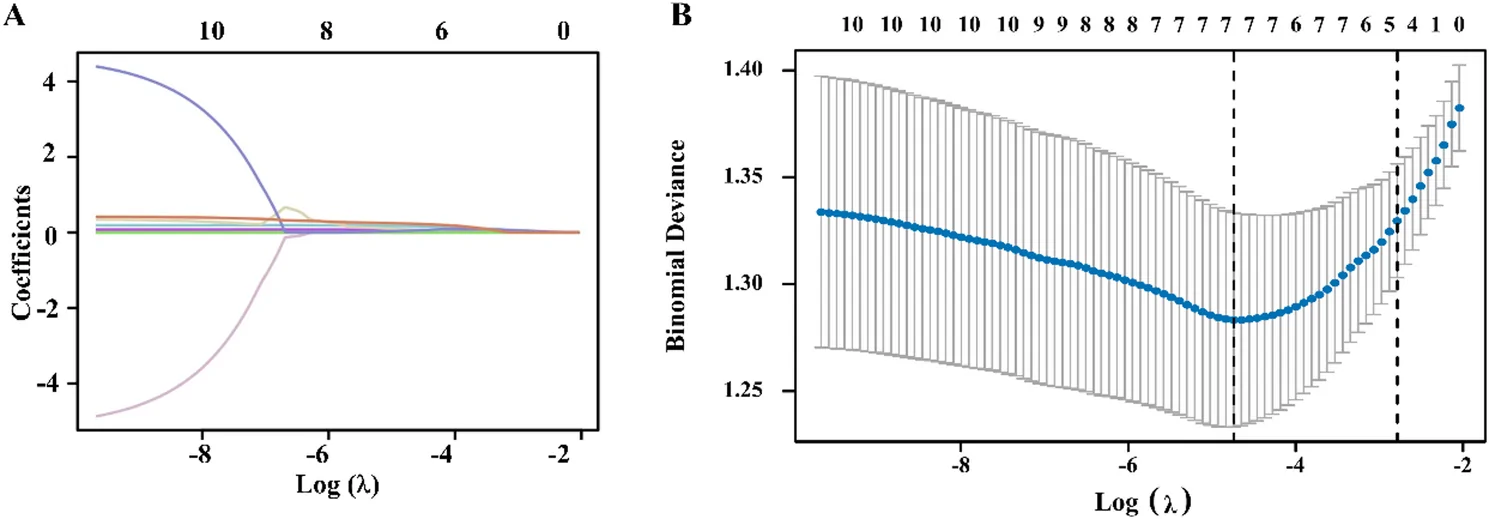
Screening of variables based on Lasso logistic regression. (**A**) The variation characteristics of the coefficient of variables; (**B**) The selection process of the optimum value of the parameter λ in the Lasso-regression model by 10-fold cross validation method, use the minimum standard and 1se (1-SE standard) of the minimum standard to draw a vertical dashed line at the optimal value

Based on the aforementioned findings, we analyzed 10 variables, including LDNs, CEA, CA153, NLR, PLR, SII, SIRI, AISI, NMLR, and NHR to screen the parameters. A Lasso regression was employed to develop a prediction model, with an optimal lambda value determined by minimizing the cross-validation error, as visualized in Fig. 3A. The 10-fold cross validation method was applied to the iterative analysis, and a model with excellent performance but minimum number of variables was obtained when λ was 0.009 (log λ= −2.046) (Fig. 3B). Finally, seven key variables were selected: LDNs, CEA, CA153, PLR, SIRI, NMLR, and NHR.

### Association between lymph node status and screening variables

**Fig. 4:**
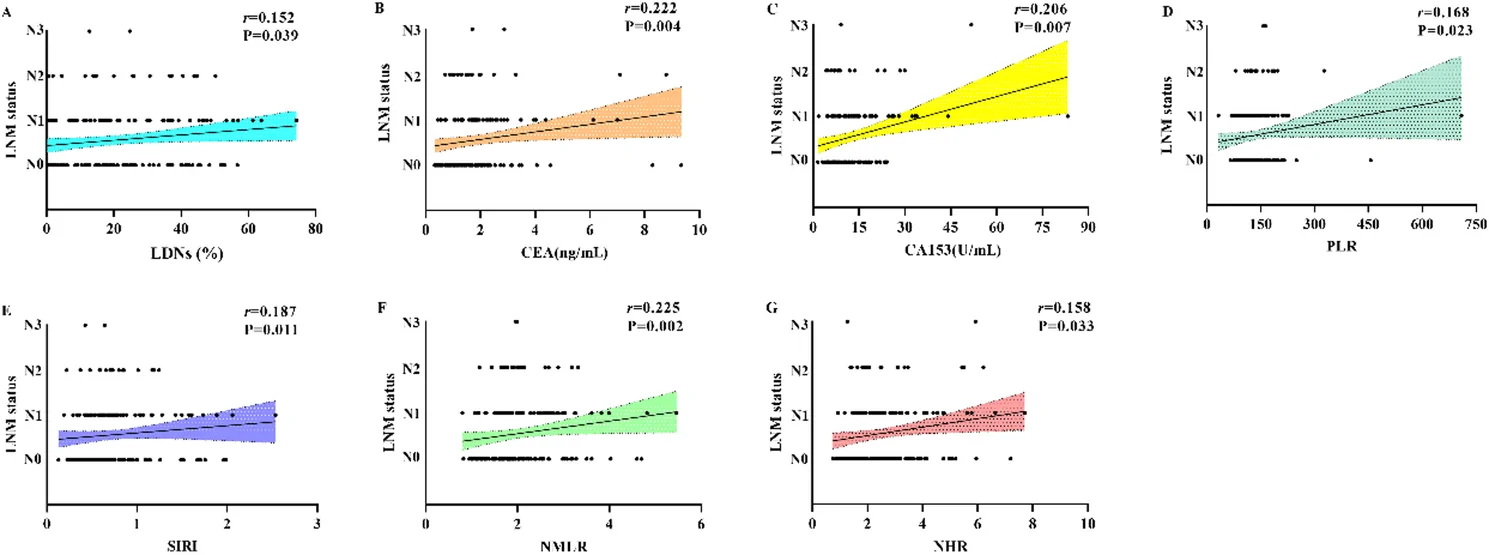
Association between lymph node status and screening variables in patients using Spearman’s analysis. Abbreviations: LDNs, low-density neutrophils; PLR, platelet-to-lymphocyte ratio; SIRI, systemic inflammation response index; NMLR, (neutrophil + monocyte)-to-lymphocyte ratio; NHR, neutrophil to HDL­C ratio

According to the lymph node status, we divided the patients into four groups: N0, N1, N2, and N3. As shown in Fig. 4, LDNs (*r* = 0.156, *P* = 0.039), CEA (*r* = 0.222, *P* = 0.004), CA153 (*r* = 0.206, *P* = 0.007), PLR (*r* = 0.168, *P* = 0.023), SIRI (*r* = 0.187, *P* = 0.011), NMLR (*r* = 0.225, *P* = 0.002) and NHR (*r* = 0.158, *P* = 0.033) were positively correlated with lymph node status.

### Diagnostic efficiency of screening variables for LNM in patients

ROC-AUC analysis was performed to evaluate the predictive value of the screening variables for the presence and severity of LNM. Table 2 lists the optimal cut-off values for each variable: LDNs at 25.27%, CEA at 1.63 ng/mL, CA153 at 11.35 U/mL, PLR at 118.49, SIRI at 0.57, NMLR at 1.67, and NHR at 1.72. The diagnostic performance of each variable was as follows: LDNs yielded an AUC of 0.590 (95% CI: 0.507–0.674), with 43.90% sensitivity and 75.51% specificity. For CEA, the AUC was 0.631 (95% CI: 0.547–0.716), accompanied by a sensitivity of 59.46% and a specificity of 65.26%. CA153 showed an AUC of 0.636 (95% CI: 0.552–0.721), with corresponding sensitivity and specificity values of 55.41% and 67.37%, respectively. In addition, PLR had an AUC of 0.584 (95% CI: 0.500–0.669; sensitivity: 68.29%; specificity: 51.96%). SIRI had an AUC of 0.615 (95% CI: 0.533–0.697; sensitivity: 80.49%; specificity: 50.98%). NMLR had an AUC of 0.636 (95% CI: 0.555–0.716; sensitivity: 81.71%; specificity: 41.18%), and NHR had an AUC of 0.600 (95% CI: 0.519–0.682; sensitivity: 84.15%; specificity: 33.33%). Further metrics, including accuracy, positive predictive value (PPV), and negative predictive value (NPV), and Youden’s index were detailed in Table 2. In addition, we have performed subgroup analyses to evaluate the clinical relevance of these cutoffs by examining their associations with clinicopathological features, as shown in the newly added Supplementary materials **Table S2**. Patients stratified by these cutoffs exhibited significant differences in lymph node status. These findings suggest that the derived cutoffs effectively identify patients at higher risk of LNM.

**Table 2:** Diagnostic efficiency of screening variables for breast cancer patients with lymph node metastasis

Factors	AUC	95%CI	Se (%)	Sp (%)	Ac (%)	PPV (%)	NPV (%)	Cutoff value	Youden’s index
LDNs (%)	0.590	0.507–0.674	43.90	75.51	60.87	58.07	62.30	25.27	0.184
CEA (ng/mL)	0.631	0.547–0.716	59.46	65.26	62.72	57.14	67.39	1.63	0.247
CA153(U/mL)	0.636	0.552–0.721	55.41	67.37	62.13	56.94	65.98	11.35	0.228
PLR	0.584	0.500–0.669	68.29	51.96	59.24	53.33	67.09	118.49	0.203
SIRI	0.615	0.533–0.697	80.49	50.98	64.13	56.90	76.47	0.57	0.315
NMLR	0.636	0.555–0.716	81.71	41.18	59.24	52.76	73.68	1.67	0.229
NHR	0.600	0.519–0.682	84.15	33.33	55.98	50.37	72.34	1.72	0.175

*AUC* area under curve, *CI* confidence interval, *Se* sensitivity, *Sp* specificity, *Ac* accuracy, *PPV* positive predictive value, *NPV* negative predictive value, *LDNs* low ⁃ density neutrophils, *CEA* carcinoembryonic antigen, *CA153* carbohydrate antigen 153, *PLR* platelet-to-lymphocyte ratio, *SIRI* the systemic inflammation response index, *NMLR* (neutrophil + monocyte)-to-lymphocyte ratio, *NHR* neutrophil to HDL­C ratio

### Association between clinicopathological features and LDNs percentage

Based on the cut-off value of 25.27% for LDNs, we divided the samples into high- and low-expression groups, with 122 patients showing a low LDNs percentage and 62 showing a high LDNs percentage. The association between the percentage of LDNs and the clinicopathological parameters were summarized in Table 3. For breast cancer samples, a low LDNs percentage was significantly more frequently observed in TNM stage I and II samples than in stage III samples (*P* = 0.005). Moreover, we noted a significant correlation between the percentage of LDNs and LNM (*P* = 0.023). However, statistical analysis revealed no significant correlation between the percentage of LDNs and age, BMI, or the positive rates of ER, PR, HER-2 and Ki-67 (all *P* > 0.05).

**Table 3:** Correlation between low-density neutrophils percentage and clinicopathological characteristics

Characteristics	Number of patients	LDNs low expression (≤ 25.27%)	LDNs high expression (> 25.27%)	*P* value
Number	184	122	62	
Age(years)				0.190
≤ 55		84	36	
> 55		38	26	
BMI (kg/m^2^)				0.864
< 18.5	2	2	0	
18.5–23.9	78	48	30	
24-27.9	76	55	21	
≥ 28.0	28	17	11	
Tumor stage				0.005**
Stage I	47	36	11	
Stage II	109	74	35	
Stage III	28	12	16	
Lymph node metastasis				0.023*
N0	102	76	26	
N1	63	36	27	
N2	17	8	9	
N3	2	2	0	
ER				0.636
Negative	55	34	21	
Positive	126	87	39	
Unknown	3	1	2	
PR				0.679
Negative	72	46	26	
Positive	107	73	34	
Unknown	5	3	2	
Her-2				0.865
Negative	23	15	8	
Positive	157	105	52	
Unknown	4	2	2	
Ki-67				0.053
≤ 30%	95	68	27	
> 30%	83	52	31	
Unknown	6	2	4	

*BMI* body mass Index, *LDNs* low ⁃ density neutrophils, *ER* estrogen receptor, *PR* progesterone Receptor, *Her-2* epidermal growth factor receptor-2. *P < 0.05, **P < 0.01

### Univariate and multivariate logistic regression analysis

LNM presence (code as 0 for absent, 1 for present) served as the dependent variable. Independent variables were dichotomized based on their optimal cut-off values: LDNs (> 25.27%), CEA (> 1.63 ng/mL), CA153 (> 11.35 U/mL), PLR (> 118.49), SIRI (> 0.57), NMLR (> 1.67), and NHR (> 1.72), with values at or below these thresholds coded as 0 and those above as 1. As shown in Table 4, these indicators were significantly associated with LNM (all *P* < 0.01). Multivariate analysis identified LDNs (OR = 2.307, *P* = 0.032), CEA (OR = 4.027, *P* < 0.001), CA153 (OR = 2.440, *P* = 0.016), PLR (OR = 2.563, *P* = 0.014), and SIRI (OR = 4.617, *P* = 0.003) as independent risk factors for LNM. Immediately after, collinearity diagnostics were performed using the variance inflation factor (VIF) for the five independent predictors retained in the final multivariate model. The VIF values were 1.18 for LDNs, 1.22 for CEA, 1.15 for CA153, 1.20 for PLR, and 1.16 for SIRI, all well below the conventional threshold of 5, indicating no significant multicollinearity.

**Table 4:** Univariate and multivariate logistic analysis in patients with lymph node metastasis

Variables	Univariate			Multivariate		
	OR	95% CI	*P* value	OR	95% CI	*P* value
LDNs (%)			0.006**			0.032*
≤ 25.27	1			1		
> 25.27	2.410	1.287–4.515		2.307	1.076–4.943	
CEA (ng/mL)			0.002**			< 0.001***
≤ 1.63	1			1		
> 1.63	2.756	1.471–5.161		4.027	1.876–8.646	
CA153 (U/mL)			0.003**			0.016*
≤ 11.35	1			1		
> 11.35	2.565	1.369–4.805		2.440	1.181–5.042	
PLR			0.006**			0.014*
≤ 118.49	1			1		
> 118.49	2.330	1.271–4.270		2.563	1.206–5.443	
SIRI			< 0.001***			0.003**
≤ 0.57	1			1		
> 0.57	3.976	2.055–7.695		4.617	1.707–12.489	
NMLR			0.001**			0.688
≤ 1.67	1			1		
> 1.67	3.006	1.534–5.892		0.823	0.319–2.126	
NHR			0.014*			0.401
≤ 1.72	1			1		
> 1.72	2.429	1.197–4.927		1.476	0.594–3.667	

*LDNs* low ⁃ density neutrophils, *CEA* carcinoembryonic antigen, *CA153* carbohydrate antigen 153, *PLR* platelet-to-lymphocyte ratio, *SIRI* the systemic inflammation response index, *NMLR* (neutrophil + monocyte)-to-lymphocyte ratio, *NHR* neutrophil to HDL­C ratio, *OR* odds ratio, *CI* confidence interval. *P < 0.05, **P < 0.01, ***P < 0.001

### Nomogram Model for Risk Assessment of breast cancer with LNM

**Fig. 5:**
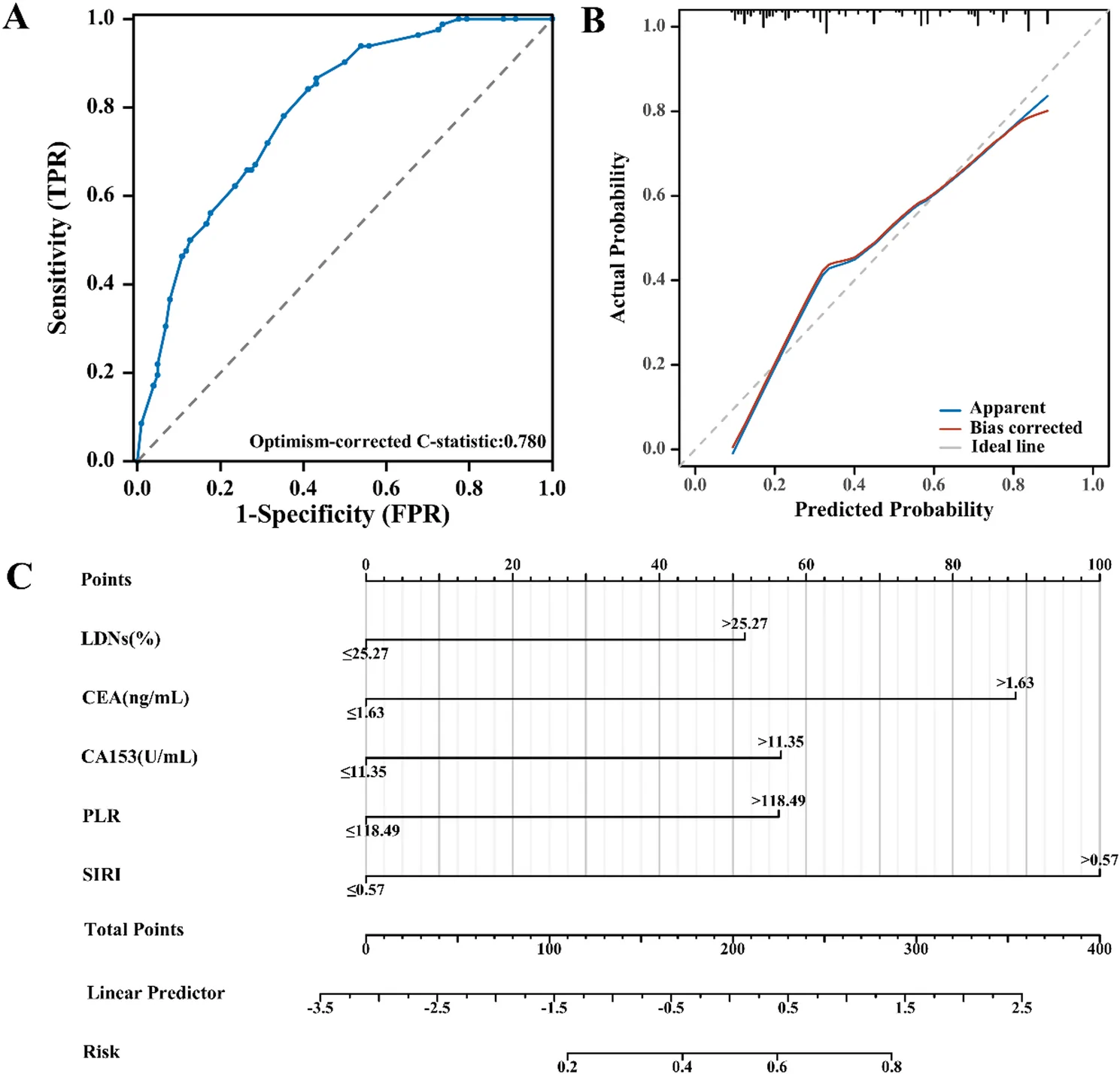
Prediction model for risk assessment of breast cancer with lymph node metastasis. (**A**) ROC curve of the combined factors; (**B**) Calibration curve of prediction model; (**C**) Nomogram of the logistic regression model. Abbreviations: AUC, Area Under Curve; CI, confidence interval; LDNs, low-density neutrophils; PLR, platelet-to-lymphocyte ratio; SIRI, systemic inflammation response index

Based on the multivariate logistic regression results, a nomogram for predicting LNM was constructed with the R package rms. The model exhibited good discriminative power, with a C-statistic of 0.791 (95% CI: 0.727–0.855), alongside a sensitivity of 86.59%, specificity of 56.86%, and an overall accuracy of 70.11%. After bootstrap optimism correction with 500 resamples, the optimism-corrected C-statistic was 0.780 (0.751–0.809), indicating minimal overfitting (optimism: 0.011) (Fig. 5A). Its calibration was further assessed through the Hosmer-Lemeshow goodness-of-fit test. There was no statistically significant difference between the predicted values of the model and actual observed values (χ^2^ = 6.420, *P* = 0.600) (Fig. 5B). The final nomogram is presented in Fig. 5C. In addition, a score of LDNs > 25.27% was 52; a score of CEA > 1.63ng/mL was 88; a score of CA153 > 11.35 U/mL was 57; a score of PLR > 118.49 was 56; a score of SIRI > 0.57 was 100. A cumulative score of 353 points corresponded to an estimated lymph node metastasis probability exceeding 80%. The relationship between total points and predicted risk is further detailed in Table 5. To facilitate clinical application, we evaluated the performance of the nomogram at different risk thresholds. The results are summarized in Table 6. At the proposed threshold of > 60% risk (total points > 224), the nomogram achieved a sensitivity of 50.0% and a specificity of 87.25%, with a PPV of 75.92% and an NPV of 68.46%. A lower threshold of > 50% (total points > 170) yielded higher sensitivity (62.19%) at the cost of lower specificity (76.47%), which may be more suitable for screening purposes.

**Table 5:** Relationship between total points and risk of lymph node metastasis for breast cancer patients

Total points	Risk of LNM (%)
< 109	< 20
109–170	20–40
170–224	41–60
224–285	61–80
> 285	> 80

*LNM* lymph node metastasis

**Table 6:** Performance of the nomogram at different risk thresholds in the training cohort

Risk threshold	Sensitivity (%)	Specificity (%)	PPV (%)	NPV (%)
> 50%	62.19	76.47	68.00	71.56
> 55%	56.10	82.35	71.88	70.00
> 60%	50.00	87.25	75.92	68.46
> 70%	30.49	93.14	78.13	62.50

*PPV* positive predictive value, *NPV* negative predictive value

### Validation of the nomogram in an independent external cohort

**Fig. 6:**
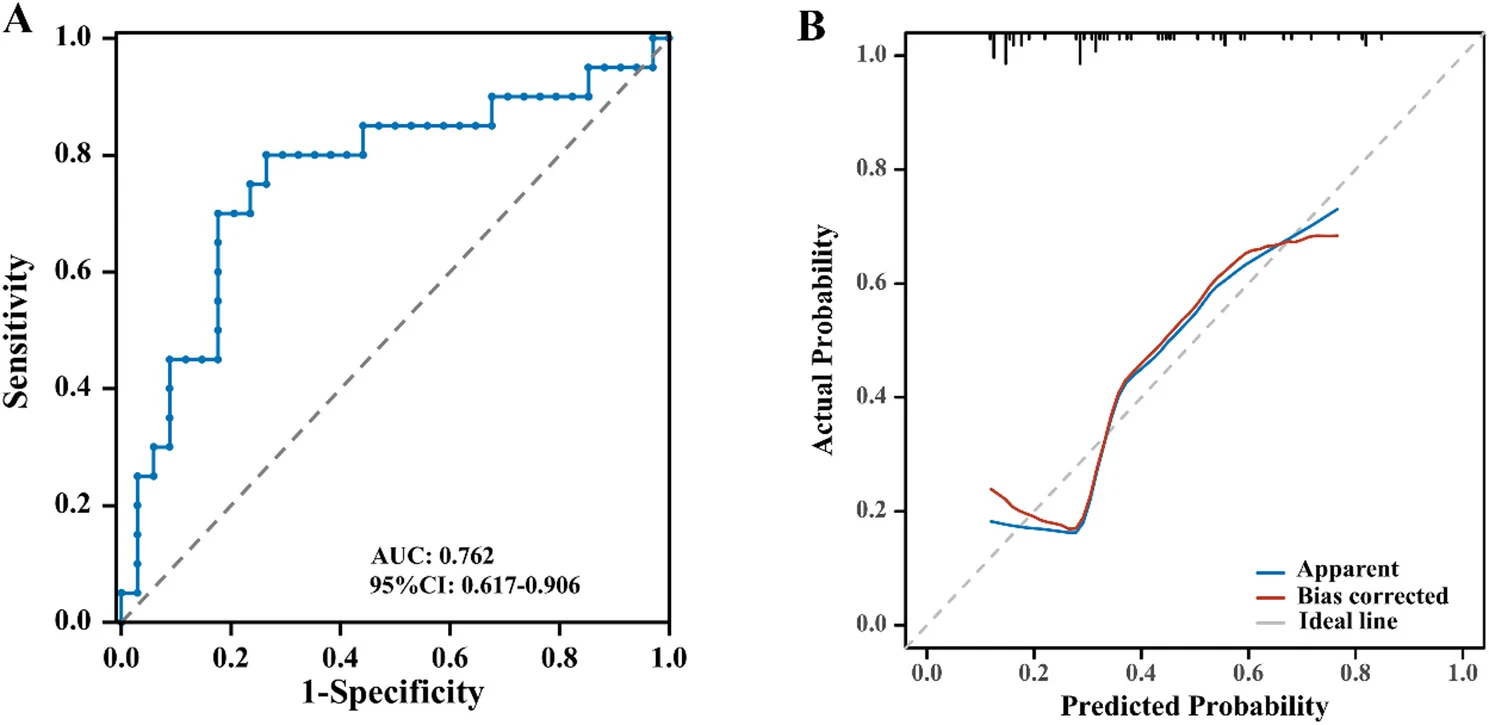
Validation of the prediction model. (**A**) ROC curve of model in the validation group. (**B**) Calibration curve of model in the validation group. AUC, area under the curve; CI, confidence interval

As for external validation cohort consisted of 54 breast cancer patients. There was no statistically significant difference in clinical data between the training group and validation group (Supplementary materials Table S3). What’s more, we have calculated the AUC, sensitivity, specificity, PPV, and NPV for each of the five binary indicators (LDNs, CEA, CA153, PLR, SIRI) in the external validation cohort (*n* = 54). These results have been added to a new Supplementary materials Table S4. And then, the ROC curves of the prediction model are displayed in Fig. 6A for the validation group (AUC = 0.762, 95%CI: 0.617–0.906). These results suggested that nomogram exhibits promising predictive capability in identifying LNM in breast cancer patients, with AUC values exceeding 0.75. Furthermore, the calibration curves also demonstrated good predictive power (Fig. 6B). The Hosmer-Lemeshow test demonstrated that *P* = 0.722 and χ^2^ = 5.331 with *P* > 0.05, indicating that the model fit was good.

## Discussion

Recent data show a 0.6%-1% annual increase in breast cancer rates from 2015 to 2019, posing a health threat [32]. Advances in understanding breast cancer’s molecular and cellular mechanisms have improved treatments, but many patients still face disease progression and recurrence [33]. Lymph nodes facilitate local metastasis, allowing tumor cells to spread. While SLN biopsy is common for assessing lymph node involvement, it is invasive, requiring skill and posing risks like longer surgery times, lymphedema of the upper limb, and numbness [2]. Early non-invasive diagnosis could reduce patient discomfort. Identifying feasible and easily accessible early effective indicators is crucial for screening patients at high risk of LNM.

LDNs, a unique neutrophils subpopulation, are garnering more attention because their abundance is associated with the development and progression of numerous disease [34, 35]. Research has revealed that LDNs are absent in the blood of healthy individuals and only appear during inflammatory and/or pathological conditions [36]. In healthy individuals, LDNs constitute approximately 5% or less of the cells isolated from PBMCs [12, 37]. Our observation indicated that the median level of LDNs in healthy control was 2.49%, representing merely 15.16% of the LDNs present in patients with breast cancer. Similarly, Saraiva et al. confirmed the near absence of LDNs in the blood of healthy individuals [38]. Furthermore, the study demonstrated that patients with metastatic breast cancer exhibit higher levels of LDNs compared to those with non-metastatic breast cancer. In our investigation, elevated LDN levels were correlated with TNM staging and LNM. Furthermore, LDNs are among the variable characteristics associated with LNM in breast cancer, supporting the notion that LDNs play a role in metastasis and progressively accumulate as cancer advances [39]. LDNs exhibit significant immunosuppressive effects and pro-tumorigenic activities, including the promotion of tumor angiogenesis, invasion, and metastasis through various mechanisms [40]. They exhibit an enhanced propensity for neutrophil extracellular trap formation (NETosis), releasing NETs that physically capture circulating tumor cells, degrade the endothelial basement membrane via associated proteases, and form physical barriers that exclude CD8⁺ T cells from tumor sites [41, 42]. Additionally, LDNs contribute to tumor progression by secreting inflammatory cytokines (e.g., IL-1β, IL-6) that foster a pro-tumor inflammatory microenvironment [15]. Functionally overlapping with polymorphonuclear myeloid-derived suppressor cells, LDNs suppress adaptive anti-tumor immunity through arginase-1-mediated L-arginine depletion, reactive oxygen species production, and PD-L1 expression, collectively impairing T cell proliferation and inducing T cell exhaustion [43]. Collectively, NETs provide the physical scaffold, cytokines remodel the microenvironment, and T cell suppression ensures immune evasion, positioning LDNs as promising therapeutic targets in metastatic disease.

Accumulating evidence has demonstrated that inflammation is a hallmark of cancer and significantly contributes to tumor progression and therapeutic outcomes [44, 45]. Inflammation is an immune response triggered when the immune system detects harmful stimuli, such as pathogens, damaged cells, or toxic substances, through pattern recognition receptors, thereby activating signaling pathways and inflammasomes, leading to the release of pro-inflammatory cytokines [46]. Currently, clinicians pay more attention to the role of CBC-derived inflammatory markers in reflecting the dynamic relationship between hematological parameters in various pathological states [47]. In addition to LDNs > 25.27%, CEA > 1.63ng/mL, CA153 > 11.35 U/mL, PLR > 118.49, and SIRI > 0.57 were also independent risk factors for LNM in our study. The optimal cutoffs for LDNs, CEA, CA153, PLR, and SIRI were all significantly associated with LNM, the primary endpoint of this study. Direct comparisons of LDNs cutoffs across studies remain limited. In non-small cell lung cancer, Arasanz et al. reported an optimal LDNs cutoff of 7.09% for predicting resistance to anti-PD-1/PD-L1 immunotherapy, which is somewhat lower than our 25.27% [48]. This difference may be attributable to variations in cancer type, disease stage (advanced NSCLC vs. early breast cancer), and clinical endpoint (immunotherapy resistance vs. lymph node metastasis). Additionally, Charoensappakit et al. observed that LDN levels exceeding 30% were associated with sepsis-induced immunosuppression [49]. Our cutoff of 25.27% lies between these two values, which seems biologically plausible, as the chronic low-grade inflammation typically seen in breast cancer might lead to a moderate elevation of LDNs, distinct from the acute elevation observed in sepsis or the context-dependent elevation in NSCLC. These findings suggest that cancer- and context-specific LDNs thresholds may be needed for optimal clinical application.

Furthermore, LDNs and PLR demonstrated additional associations with advanced TNM stage, suggesting that these two biomarkers may reflect broader disease progression beyond nodal involvement. The lack of significant association between CEA, CA153, SIRI and TNM stage may be attributed to the limited sample size or their specificity to nodal status rather than overall tumor burden. The PLR, derived from platelet and lymphocyte markers, serves as a biomarker for systemic inflammation and has diagnostic and prognostic value in cancer. Platelets, beyond their role in hemostasis, regulate the tumor microenvironment and interact with immune cells, affecting disease progression [50]. Lymphocytes are integral to antitumor immunity and tumor-associated immune responses [51]. In breast cancer, PLR may serve as a potential biomarker for the early diagnosis, with an AUC value of 0.665, 61.90% sensitivity, and 64.29% specificity [52]. High PLR is linked to cervical LNM in salivary gland adenoid cystic carcinoma and predicts overall survival [53]. SIRI is a newly recognized marker that reflects the interplay between inflammatory and immune states by integrating counts of peripheral blood neutrophils, monocytes, and lymphocytes [54]. Neutrophils, as key players in inflammation, manipulate the microenvironment to promote tumor growth by suppressing immune responses and releasing cytokines that influence lymphocytes and monocytes [55]. Monocytes, upon reaching tumors, interact with damaged endothelial cells to secrete proinflammatory cytokines and adhesion molecules, further aiding tumor progression [56]. Inflammation often alters hematological parameters, including neutrophil, lymphocyte, and monocyte counts, leading to increased SIRI values. Several studies have identified SIRI as a diagnostic and prognostic biomarker for various tumors. In our previous study, SIRI could be used as a screening biomarker in the diagnosis of gastric cancer, especially in the early stages of male patients [57]. In advanced rectal cancer, a high SIRI independently predicts poor overall and disease-free survival in patients receiving neoadjuvant chemoradiotherapy [58]. However, the remaining 5 screened variables (NLR, SII, AISI, NMLR, and NHR) were not included in the model, possibly due to a lack of independent statistical significance after adjustment or redundancy with the selected indicators. Notably, PLR and SIRI are composite inflammatory indices that integrate neutrophil, lymphocyte, and monocyte counts, which may account for the predictive information carried by NLR, SII, AISI, and NMLR. Similarly, NHR may share overlapping inflammatory signals with the selected indices. Thus, the final five-variable model achieves a balance between parsimony and predictive performance, incorporating both tumor-specific biomarkers (CEA, CA15-3) and complementary inflammatory indicators (LDNs, PLR, SIRI). In addition, collinearity diagnostics showed that all VIF values were below 1.23, indicating no significant multicollinearity among the selected predictors. These findings further justify the exclusion of NMLR and NHR, as their non-significance in the multivariate model was not attributable to collinearity with the retained predictors.

Nowadays, emerging evidence suggests that immune-related molecules play crucial roles in breast cancer progression. Through systematic pan-cancer analyses with breast cancer validation, Guo’s research group has identified several key immune-associated biomarkers: BYSL as a prognostic indicator correlated with MDSC infiltration [59], CORO1A as a diagnostic and prognostic marker involved in B cell receptor pathways [60], CENPN as a molecule with significant immunological functions [61], and CLIC6 as a protective factor associated with tumor immune microenvironment and favorable outcomes [62]. These findings underscore the value of immune-based biomarkers in breast cancer. Building upon this evidence, our study focused on circulating LDNs and inflammatory markers, which represent readily accessible immune parameters for clinical application in patients with LNM.

Immediately after, we established a nomogram for breast cancer with LNM. The C-statistic of the model evaluation was 0.791. It is worth noting that a small proportion of cases (9 cases, 4.9%) had missing data for ER, PR, HER-2, or Ki-67. Given the low rate and random distribution of missingness, pairwise exclusion was considered appropriate. Furthermore, a sensitivity analysis excluding these cases yielded comparable results (C-statistic: 0.790 vs. 0.791 in the primary analysis), indicating that the missing data are unlikely to have biased our conclusions. Calibration curve analysis further confirmed the excellent discriminatory ability and clinical utility of the nomogram. A reliable multivariate prediction model, particularly a visual nomogram, enables clinicians to quickly identify the target population and helps clinicians make accurate and wise clinical decisions [63, 64]. For potential clinical application, a risk threshold of > 60% (total points > 224) may be considered to guide sentinel lymph node biopsy. At this threshold, the nomogram yielded a specificity of 87.25% and a sensitivity of 50.00%, with a PPV of 75.92%. This threshold appears to prioritize specificity, which may help reduce unnecessary invasive procedures. Alternatively, a lower threshold (> 50% or > 55%) could be considered when higher sensitivity is preferred, depending on the clinical context. For instance, the > 50% threshold offered a sensitivity of 62.19% with a specificity of 76.47%, which may be more suitable for screening purposes. Conversely, the > 70% threshold provided high specificity (93.14%) but with a substantial loss of sensitivity (30.49%), which may be less practical for routine use. A possible clinical workflow might be: (1) calculate total points using the nomogram; (2) convert total points to predicted LNM risk using Table 5; (3) if risk exceeds 60%, SLNB could be considered; (4) if risk is 60% or lower, observation or less invasive evaluation might be an option. These findings should be interpreted with caution, and prospective validation in independent cohorts would be needed before routine clinical use.

In the external validation cohort, the individual biomarkers showed moderate to low discriminative ability (AUC range: 0.529–0.684), with CA153 demonstrating the highest specificity (79.41%) and SIRI the highest sensitivity (75.00%). However, the combined nomogram achieved an AUC of 0.762 and good calibration, substantially outperforming any single indicator. This highlights the complementary value of integrating multiple biomarkers for LNM prediction. These findings provide preliminary evidence supporting the generalizability of the nomogram. Although the external validation cohort was temporally independent, its relatively small size may introduce some uncertainty into the performance estimates. However, the validation AUC was close to that of the training cohort, which suggests that the model has reasonable generalizability. Given that both cohorts were from a single institution, larger prospective multicenter studies are required to further establish the clinical utility of the nomogram. The nomogram is intended as a complementary tool for preoperative assessment of LNM risk in breast cancer patients. It may assist in clinical decision-making by identifying high-risk patients who could benefit from sentinel lymph node biopsy or more extensive axillary dissection, while potentially reducing unnecessary invasive procedures in low-risk individuals. The model is not intended to replace imaging or pathological diagnosis but rather to serve as an adjunctive risk stratification tool. Although these applications are promising, prospective studies are warranted to validate the nomogram’s clinical utility and its impact on patient outcomes before routine implementation.

Several limitations should be considered in interpreting our findings. First, this was a retrospective single-institution study, future research involving multi-institution and a larger patient population is warranted, aiming to obtain more reliable results for clinical application. Second, the mechanisms underlying LDN-mediated promotion of LNM in breast cancer patients, including the release of inflammatory cytokines such as TNF-α and IL-6 and the formation of NETs, were not explored in the present study. These represent key priorities for our future mechanistic research. Third, several potential confounders, including tumor subtype, menopausal status, and systemic inflammation, were not fully controlled in this study. Although the association between LDNs and LNM remained significant in preliminary subgroup analyses, multi-institution and larger cohorts are warranted to adjust for these factors and further validate the robustness of our findings.

## Conclusions

The present study revealed elevated levels of LDNs in the peripheral blood of breast cancer patients, particularly in those with LNM. LDNs > 25.27%, CEA > 1.63ng/mL, CA153 > 11.35 U/mL, PLR > 118.49, and SIRI > 0.57 were identified as independent risk factors for LNM. The nomogram integrating these biomarkers may assist in LNM risk assessment in breast cancer patients, and could potentially be developed into an online web tool after further validation.

## Supplementary Information

Supplementary Material 1: Basic characteristics of 184 patients with breast cancer were shown in Supplementary material Table S1.

Supplementary Material 2: Association between cutoff-defined groups and clinicopathological characteristics in Supplementary material Table S2.

Supplementary Material 3: Comparison of clinicopathological characteristics between the training group and validation group in Supplementary Table S3

Supplementary Material 4: Performance of individual cutoffs in the external validation cohort in Supplementary Table S4.

## Data Availability

The data supporting the results of this study are not publicly available due to patient privacy and ethical restrictions, but are available from the corresponding author upon reasonable request.
