# Supplementary material for: The clinical value of the combined detection of circulating low ⁃ density neutrophils and inflammatory markers in breast cancer with lymph node metastasis
Source: BMC Cancer. 2026 Jun 23;26:1061. doi: 10.1186/s12885-026-16384-6 (PMC13548714; doi:10.1186/s12885-026-16384-6)
Supplement: Supplementary file 1 [file 12885_2026_16384_MOESM1_ESM.docx]

**Table S1.** Basic characteristics of 184 patients with breast cancer

| Characteristic | Patients | Percentage (%) |
| --- | --- | --- |
| Treatment methods after admission |  |  |
| Surgery | 154 | 83.70 |
| Neoadjuvant chemotherapy | 30 | 16.30 |
| Surgery |  |  |
| Modified radical mastectomy + SLNB | 109 | 70.78 |
| Modified radical mastectomy + SLNB+ALNR | 36 | 23.38 |
| Breast conserving surgery+ SLNB | 1 | 0.65 |
| Local enlarged mass resection | 7 | 4.54 |
| Total mastectomy | 1 | 0.65 |
| Tumor size (cm) |  |  |
| <1 | 6 | 3.90 |
| 1-2 | 63 | 40.91 |
| 2-3 | 44 | 28.57 |
| ≥3 | 41 | 26.62 |
| Lymph node status |  |  |
| positive | 82 | 44.57 |
| negative | 102 | 55.43 |
| TNM stage |  |  |
| I | 47 | 25.54 |
| II | 109 | 59.24 |
| III | 28 | 15.22 |

SLNB: Sentinel lymph node biopsy; ALNR: axillary lymph node resection
