# Supplementary material for: The clinical value of the combined detection of circulating low ⁃ density neutrophils and inflammatory markers in breast cancer with lymph node metastasis
Source: BMC Cancer. 2026 Jun 23;26:1061. doi: 10.1186/s12885-026-16384-6 (PMC13548714; doi:10.1186/s12885-026-16384-6)
Supplement: Supplementary file 2 [file 12885_2026_16384_MOESM2_ESM.docx]

**Table S2.** Association between cutoff-defined groups and clinicopathological characteristics

| Characteristic | LDNs  (>25.27%) | CEA  (>1.63 ng/mL) | CA153  (>11.35U/mL) | PLR  (>118.49) | SIRI  (>0.57) | NMLR  (>1.67) | NHR  (>1.72) |
| --- | --- | --- | --- | --- | --- | --- | --- |
| LNM(Y) |  |  |  |  |  |  |  |
| Yes, n (%) | 36/62(58.06) | 49/85(57.65) | 46/80(57.50) | 56/105(53.33) | 65/115(56.52) | 66/125(52.80) | 68/136(50.00) |
| No, n (%) | 46/122(37.70) | 33/99(33.33) | 36/104(34.62) | 26/79(32.91) | 17/69(24.64) | 16/59(27.12) | 14/48(29.17) |
| *P* value | 0.009** | <0.001*** | 0.002* | 0.006** | <0.001*** | <0.001*** | 0.013* |
| TNM stage (III) |  |  |  |  |  |  |  |
| Yes, n (%) | 16/62(25.81) | 15/85(17.65) | 14/80(17.50) | 21/105(20.00) | 20/115(17.39) | 22/125(17.60) | 20/136(14.71) |
| No, n (%) | 12/122(9.84) | 13/99(13.13) | 14/104(13.46) | 7/79(8.86) | 8/69(11.59) | 6/59(10.17) | 8/48(16.67) |
| *P* value | 0.004** | 0.395 | 0.450 | 0.037* | 0.289 | 0.190 | 0.745 |

LNM: lymph node metastasis; TNM: tumor-node-metastasis; LDNs: low ⁃ density neutrophils; CEA: carcinoembryonic antigen; CA153: carbohydrate antigen 153; PLR: platelet-to-lymphocyte ratio; SIRI: the systemic inflammation response index; NMLR: (neutrophil + monocyte)-to-lymphocyte ratio; NHR: neutrophil to HDL­C ratio. **P*<0.05, ***P*<0.01, ****P*<0.001.
