# Supplementary material for: The clinical value of the combined detection of circulating low ⁃ density neutrophils and inflammatory markers in breast cancer with lymph node metastasis
Source: BMC Cancer. 2026 Jun 23;26:1061. doi: 10.1186/s12885-026-16384-6 (PMC13548714; doi:10.1186/s12885-026-16384-6)
Supplement: Supplementary file 3 [file 12885_2026_16384_MOESM3_ESM.docx]

**Table S3.** Comparison of clinicopathological characteristics between the training group and validation group

|  | Characteristics | Training group  (n=184) | | Validation group  (n=54) | *P* value |
| --- | --- | --- | --- | --- | --- |
|  | Age(years) | 52(45, 58) | 55(46, 61) | | 0.064 |
|  | BMI (kg/m^2^) | 24.39(22.05, 26.65) | 23.97(22.60, 25.52) | | 0.338 |
|  | LDNs (%) | 16.43(4.75, 34.63) | 10.51(5.07, 35.64) | | 0.610 |
|  | CEA (ng/mL) | 1.53(0.94, 2.21) | 1.54(1.13, 2.20) | | 0.278 |
|  | CA153 (U/mL) | 10.10(7.04, 14.80) | 9.16(5.99, 11.38) | | 0.069 |
|  | CA125 (U/mL) | 9.88 (7.33, 12.50) | 11.20(7.18, 15.10) | | 0.415 |
|  | NLR | 1.69(1.35, 2.22) | 1.81(1.40, 2.48) | | 0.179 |
|  | PLR | 123.74(100.40, 152.44) | 136.62(102.51, 160.78) | | 0.157 |
|  | LMR | 4.66(3.79, 5.45) | 4.95(4.02, 6.07) | | 0.219 |
|  | SII | 384.29(286.18, 477.95) | 396.15(281.28,638.93) | | 0.260 |
|  | PNI | 53.83(50.44, 57.15) | 53.90(51.34, 56.53) | | 0.867 |
|  | SIRI | 0.65(0.48, 0.85) | 0.59(0.44, 0.91) | | 0.741 |
|  | AISI | 150.51(106.80, 195.88) | 138.82(96.21, 232.24) | | 0.947 |
|  | NMLR | 1.90(1.59, 2.51) | 2.00(1.58, 2.76) | | 0.283 |
|  | NHR | 2.28(1.72, 3.10) | 2.32(1.76, 3.33) | | 0.413 |
|  | MHR | 0.30(0.22, 0.39) | 0.26(0.22, 0.35) | | 0.107 |
|  | LHR | 1.38(1.02, 1.81) | 1.32(0.99, 1.66) | | 0.362 |
|  | PHR | 168.75(137.51, 219.37) | 176.33(144.56, 210.92) | | 0.751 |
|  | ER |  |  | | 0.053 |
| Negative | | 50(27.17) | 10(18.52) | |  |
|  | Positive | 131(71.20) | 40(74.07) | |  |
|  | Unknown | 3(1.63) | 4(7.41) | |  |
|  | PR |  |  | | 0.057 |
| Negative | | 72(39.13) | 15(27.78) | |  |
|  | Positive | 107(58.15) | 35(64.81) | |  |
|  | Unknown | 5(2.72) | 4(7.41) | |  |
|  | Her-2 |  |  | | 0.323 |
|  | Negative | 23(12.50) | 13(24.07) | |  |
|  | Positive | 157(85.33) | 37(68.52) | |  |
|  | Unknown | 4(2.17) | 4(7.41) | |  |
|  | Ki-67 |  |  | | 0.836 |
|  | ≤30% | 81(44.02) | 27(50.00) | |  |
|  | >30% | 97(52.72) | 23(42.59) | |  |
|  | Unknown | 6(3.26) | 4(7.41) | |  |

BMI: body mass Index; LDNs: low ⁃ density neutrophils; CEA: carcinoembryonic antigen; CA125: carbohydrate antigen 125; CA153: carbohydrate antigen 153; NLR: neutrophil-to-lymphocyte ratio; PLR: platelet-to- lymphocyte ratio; LMR: lymphocyte-to-monocyte ratio; SII: systemic immune- inflammation index; PNI: prognostic nutritional index; SIRI: the systemic inflammation response index; AISI: aggregate index of systemic inflammation; NMLR: (neutrophil + monocyte)-to-lymphocyte ratio; NHR: neutrophil to HDL­C ratio; MHR: monocyte to HDL­C ratio; LHR: lymphocyte to HDL-C ratio; PHR: platelet to HDL-C ratio; ER: estrogen receptor; PR: progesterone Receptor; Her-2: epidermal growth factor receptor-2. The measurement data were expressed as the median and quartile (25%, 75%), and the enumeration data were expressed as frequency and rate (%).
