# Supplementary material for: The clinical value of the combined detection of circulating low ⁃ density neutrophils and inflammatory markers in breast cancer with lymph node metastasis
Source: BMC Cancer. 2026 Jun 23;26:1061. doi: 10.1186/s12885-026-16384-6 (PMC13548714; doi:10.1186/s12885-026-16384-6)
Supplement: Supplementary file 4 [file 12885_2026_16384_MOESM4_ESM.docx]

**Table S4** Performance of individual cutoffs in the external validation cohort (n=54)

| Factors | Cutoff | Se  (%) | Sp  (%) | PPV  (%) | NPV  (%) | AUC |
| --- | --- | --- | --- | --- | --- | --- |
| LDNs (%) | >25.27 | 45.00 | 73.53 | 50.00 | 69.44 | 0.593 |
| CEA (ng/mL) | >1.63 | 50.00 | 55.88 | 40.00 | 65.52 | 0.529 |
| CA153(U/mL) | >11.35 | 45.00 | 79.41 | 56.25 | 71.05 | 0.622 |
| PLR | >118.49 | 70.00 | 44.11 | 42.42 | 71.43 | 0.571 |
| SIRI | >0.57 | 75.00 | 61.76 | 53.57 | 80.77 | 0.684 |

Se: sensitivity; Sp: specificity; PPV: positive predictive value; NPV: negative predictive value; AUC: area under curve; LDNs: low ⁃ density neutrophils; CEA: carcinoembryonic antigen; CA153: carbohydrate antigen 153; PLR: platelet-to-lymphocyte ratio; SIRI: the systemic inflammation response index.
